# Sodium Content and Sodium Intake Contributions of Store-Bought and Restaurant-Prepared Foods in Their As-Eaten Form: National Health and Nutrition Examination Survey, 2009–2018

**DOI:** 10.1016/j.cdnut.2024.104455

**Published:** 2024-09-02

**Authors:** Debra R Keast, Patricia M Guenther

**Affiliations:** 1 Food & Nutrition Database Research, Bangor, PA, United States; 2 Guenther Consulting, Salt Lake City, UT, United States

**Keywords:** sodium reduction, sodium content, NHANES, What We Eat in America (WWEIA) food categories, food category contributions, dietary intake, food sources, restaurant food consumption, food combinations, sandwiches

## Abstract

**Background:**

Guidance from the United States Food and Drug Administration (FDA) includes targets for the food industry to voluntarily reduce the sodium content (mg/100 g) of packaged, processed, and prepared foods sold by stores and restaurants. Assessments of sodium intake by the United States population are needed to inform sodium-reduction efforts.

**Objectives:**

The objectives of this study were to assess the sodium content and sodium intake contributions of categories and subgroups of foods obtained from stores and restaurants and determine sodium intake reductions that would be achieved by meeting FDA targets.

**Methods:**

Analyses used dietary data from the National Health and Nutrition Examination Survey, What We Eat in America (WWEIA), 2009–2018, to assess sodium in foods consumed by the United States population aged 2 y or older. Data describing where foods were obtained were used to identify store-bought and restaurant-prepared foods. Combination codes were used to group foods, such as separate salad ingredients, which were eaten together. Foods in their as-eaten form were then classified into WWEIA food categories and subgroups corresponding to FDA targets. Sample-weighted estimates generated by SUDAAN analyses were used to calculate projected sodium intake reductions.

**Results:**

Store-bought, restaurant-prepared, and other foods contributed 62%, 26%, and 12%, respectively, of sodium in United States diets. Top-ranked food category contributors of sodium included sandwiches, tortilla products, pizza, poultry, soups, and breads. Subgroups of these categories contributing the most sodium included store-bought lunchmeat sandwiches and hotdogs, restaurant-prepared burgers, store-bought and restaurant-prepared tacos/burritos, restaurant-prepared pizza with meat, and store-bought white/wheat bread. Meeting the FDA targets for these subgroups achieved the highest projected sodium intake reductions.

**Conclusions:**

Reductions of sodium in widely consumed foods, such as luncheon-meat sandwiches and restaurant-prepared pizza, have the greatest impact on reducing sodium intake by the United States population. These findings could be used by restauranteurs, food manufacturers, policymakers and regulators, and clinical practitioners to inform sodium-reduction efforts.

## Introduction

In 2007–2008, the mean sodium intake by the United States population of ∼3400 mg/d was well above the recommended level of <2300 mg/d [1]; therefore, the National Academy of Medicine, formerly the Institute of Medicine, recommended strategies that federal agencies, such as the Centers for Disease Control and Prevention and USDA, should use to promote sodium-reduction efforts, reduce the prevalence of hypertension, and improve public health [2]. A small study that was later replicated found only small amounts (<15%) of sodium were inherent or naturally occurring in foods; and although some sodium (∼10%) was added using saltshakers when foods were prepared or eaten at home, most sodium (>70%) was added when foods were prepared by food manufacturers, food retailers, or the foodservice industry [3,4]. This suggested that consumers would not reduce their sodium intake substantially unless the sodium content of the food supply was reduced. Therefore, one strategy the Institute of Medicine recommended was that an agency with regulatory authority, such as the United States Food and Drug Administration (FDA), should issue guidance to facilitate the reduction of salt (i.e., sodium chloride) and other sodium-containing additives, such as sodium phosphate, which the food industry adds to foods [2].

In 2016, in draft guidance for the food industry, the FDA proposed target values to reduce the total sodium content (i.e., milligrams of total sodium, including naturally occurring and added sodium, in each 100-g portion) of packaged, processed, or prepared foods that existed in the food supply at baseline in 2010 [5]. The Centers for Disease Control and Prevention and the USDA Food Surveys Research Group (FSRG) analyzed NHANES dietary data and found that, in 2007–2008, foods obtained from stores (including grocery and other stores) contributed 68% of dietary sodium, and another 25% came from full service and quick-serve restaurant foods [6]. Their additional findings suggested that restaurant-prepared foods contained more sodium than prepared foods or ingredients obtained from stores. Therefore, the FDA sodium reduction guidance applies to prepared foods obtained from both stores and restaurants as well as to ingredients of home-prepared foods obtained from stores [5].

Sodium content reductions will have a greater impact on sodium intake reduction when top contributors of dietary sodium, as opposed to foods with lower sodium content or less frequently consumed sodium-rich foods, are identified and predominantly targeted. NHANES 2007–2008 analyses conducted by the USDA FSRG identified categories of widely consumed foods that contributed the most dietary sodium [6]. To reduce sodium intake from these top contributors, FDA proposed reductions from the baseline sodium content of 16 categories and 163 subgroups of foods [5]. Packaged food and restaurant chain sales volumes were considered when FDA conducted their baseline sodium content assessment. Foods containing mostly inherent sodium and foods containing added sodium that were infrequently consumed were not targeted.

Assessments of sodium in United States diets are needed to help develop sodium-reduction strategies, track progress, and set priorities for the achievement of FDA targets that would most effectively reduce sodium intake by the United States population. Although sodium intakes from portions of the diet obtained from separate sources, such as stores and restaurants, and food category contributions from all sources have been reported [6,7], food category contributions from separate sources are needed. Separate sodium content estimates are needed to determine whether restaurant-prepared foods contain more sodium than prepared foods or ingredients obtained from stores, and because different FDA targets were determined for packaged and restaurant foods [5].

Therefore, the objectives of this study were as follows: *1*) to determine the separate sodium intake contributions of categories of foods obtained from stores and restaurants; *2*) to compare the sodium content of store-bought foods with the sodium content of restaurant-prepared foods; *3*) to identify sodium contributions of subgroups having the greatest impact on determining the category’s sodium content value; and *4*) to determine which potential FDA target achievements would have the greatest impact on reducing sodium intake by the United States population.

## Methods

### Analytic sample

This assessment of sodium in foods consumed by the United States population aged 2 y or older was a secondary analysis of dietary data from the combined NHANES, What We Eat in America (WWEIA), 2009–2018. NHANES is an on-going, nationally representative, cross-sectional survey using a stratified multistage probability sample design to monitor the health and nutrition status of the noninstitutionalized United States population [8]. Data sets combined for this study included NHANES, 2009–2010, 2011–2012, 2013–2014, 2015–2016, and 2017–2018 [9]. The WWEIA dietary component of NHANES provides data from two 24-h recall dietary interviews. Food composition data, including sodium content (mg/100 g) values, from the USDA Food and Nutrient Database for Dietary Studies (FNDDS) corresponding to each survey cycle [[10], [11], [12], [13], [14]] were used by USDA to calculate intakes of nutrients, including sodium (milligrams per day), from consumed amounts (grams per day) of foods reported by participants. The first day of dietary intake data can be used to provide unbiased estimates of the population’s mean daily intakes [15]. Mean intake estimates were determined using dietary day 1 sample weights and complete, reliable, 24-h dietary intakes that were self-reported or proxy-reported during day 1 dietary interviews by the sample of the United States population aged 2 y or older in 2009–2018 (*n* = 40,082). Written informed consent was obtained from all participants or their parents/caretakers, and the Research Ethics Review Board of the National Center for Health Statistics approved the survey protocol. Public-use data exclude personal identifiers; therefore, further institutional review of their secondary analysis was not required.

### Food categories

For each WWEIA cycle released since 2007–2008, USDA formed mutually exclusive categories of similar FNDDS foods using a food classification system known as the WWEIA food categories [16]. If a single FNDDS code for a food combination, such as a sandwich, did not already exist in the database, each component of the combination, such as bread or luncheon-meat consumed as a sandwich, was assigned a separate FNDDS code. If a reported food in its as-eaten form had been represented by multiple FNDDS food codes, components of the reported food had been disaggregated and classified into separate WWEIA food categories. In this study, and in recent research conducted by the USDA FSRG, codes for 15 types of food combinations were used to combine and reclassify multiple components of reported foods to WWEIA food categories that represent the combined foods [17]. The “type of combination” codes include the following: (0), noncombination food; (1), beverage with additions; (2), cereal with additions; (3), bread or baked products with additions; (4), salads; (5), sandwiches; (6), soups; (7), frozen meals; (8), ice cream or frozen yogurt with additions; (9), dried beans or vegetables with additions; (10), fruit with additions; (11), tortilla products; (12), meat, poultry, fish; (13), lunchables; (14), chips with additions; and (90), other combination types.

The analytical approach of this study involves the classification of foods into categories to determine mean sodium intake contributed by the intake of foods in the categories that were consumed by the United States population. The food category contribution, otherwise known as the population proportion or percentage of total sodium, is defined as the percentage of the population’s total dietary sodium intake that was contributed by foods in a category [18,19]. This estimate is determined as a ratio of the mean sodium intake contributed by the food category to the mean sodium intake contributed by the total diet. To ensure that the food categories were consistent over all combined survey years and consistent with previous research, as shown in Supplemental Table 1, some of the WWEIA food categories were grouped together to replicate the 104 food categories used to determine the “top ten” food sources of sodium in 2007–2008 [6].

Store-bought, restaurant-prepared, and other foods were classified using the “source of food” variable, which indicates the place where most if not all ingredients or components of each reported food in its as-eaten form were obtained. Stores include grocery stores, supermarkets, convenience stores, and unknown types of stores. Restaurants include full-service restaurants with a waiter/waitress, quick-serve restaurants selling fast food or pizza, bars/taverns/lounges, and unknown types of restaurants. Many “other” sources, such as K-12 schools, child/adult care centers, meals on wheels, and adult congregate meal programs, follow federal requirements for sodium limits; therefore, other foods were not separately analyzed in this study.

### Statistical analysis

Mean sodium intake (milligrams per day) and the population proportion (percentages) of total sodium contributed by categories were determined and ranked separately for the total diet and for foods obtained from stores and restaurants. The sodium contributions of each category and source were also expressed as percentages of the column totals—that is, total sodium from each source—and as percentages of the row totals—that is, total sodium from each category. Column percentages were then compared with row totals and row percentages with column totals.

The focus of this study was to compare the sodium content of store-bought and restaurant-prepared foods; therefore, further analysis was limited to food categories contributing population proportions of total sodium that were ≥0.5% when obtained from each separate source. The sodium content (milligrams per 100 g) of a food category composite consumed by the population was determined by the ratio of the mean sodium intake (milligrams per day) and mean food intake (100 g per day) contributions of the category. Mean sodium intake (milligrams per day), mean food intake (grams per day), population proportions (%), and the sodium content (milligrams per 100 g) ratio were also determined for subgroups of the selected categories of foods obtained from stores and restaurants.

Sample-weighted data were analyzed using SUDAAN Release 11.0.3 (RTI International, 2018) [20] to adjust the standard errors of the mean and ratio estimates for the sample design. PROC DESCRIPT of SUDAAN was used to determine mean sodium intake (milligrams per day), mean food intake (grams per day), and standard errors of the mean estimates. PROC RATIO of SUDAAN was used to determine population proportions (% of total sodium), the sodium content (milligrams per 100 g) ratio, and standard errors of the ratio estimates. Confidence intervals were determined from the standard errors of the mean and ratio estimates to test the statistical significance of differences.

Supplemental Methods provide more detailed descriptions of the statistical analysis, including methods used to determine and interpret subgroup contributions. As described in Supplemental Methods and Supplemental Table 2, subgroups of the selected categories were mapped to the FDA guidance categories to identify the appropriate sodium content targets that apply to the foods in each subgroup. Sample-weighted mean food intake (grams per day) and sodium content (milligrams per 100 g) estimates generated by SUDAAN analyses were used to calculate projected sodium intake reductions that would be achieved by meeting each subgroup’s sodium content target. Projected sodium intake reductions were determined by the product of mean food intake and positive differences between the sodium content and applicable target values. If the sodium content ratio was less than the target and the difference was negative, the projected reduction was set to zero. Category reductions were determined by summing the subgroup reductions.

## Results

**FIGURE 1:**
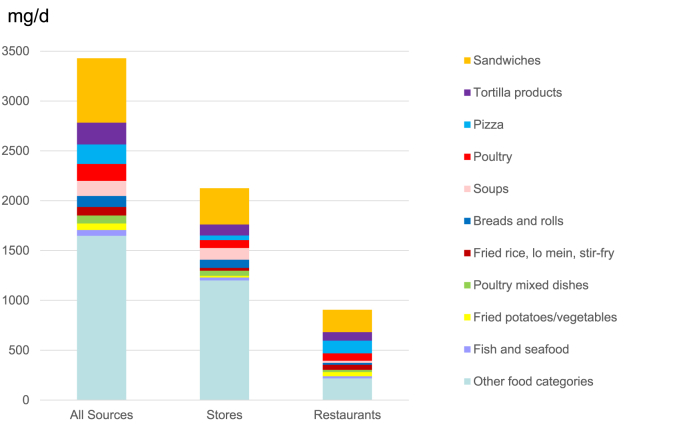
Mean sodium intake (mg/d) contributed by foods from all sources, stores, and restaurants in selected categories of foods in their as-eaten form: NHANES 2009–2018 (*n* = 40,082). Categories were selected if foods from stores and foods from restaurants both contributed ≥0.5% of total sodium.

Foods obtained from stores, restaurants, and other sources contributed 62%, 26%, and 12%, respectively, of sodium in the diets of the United States population aged 2 y or older in 2009–2018 (Figure 1). Among 16 food categories contributing the most sodium, 10 (highlighted) categories met the inclusion criterion in that store-bought and restaurant-prepared foods both contributed ≥0.5% of total sodium (Supplemental Table 3). These 10 categories contributed 52% of total sodium, and all other categories contributed 48% of total sodium (Figure 1). Foods from stores, restaurants, and other sources in the 10 categories contributed 27%, 20%, and 5% of total sodium, respectively, while foods from stores, restaurants, and other sources in the 94 other categories contributed 35%, 6%, and 7% of total sodium, respectively.

For 7 of the 10 (highlighted) categories, namely sandwiches, tortilla products, pizza, poultry, fried rice/lo-mein/stir-fry, fried potatoes/vegetables, and fish/seafood, sodium contributions of store-bought forms were lower (*P* < 0.05), and sodium contributions of restaurant-prepared forms were higher (*P* < 0.05) than the average (Table 1). Store-bought and restaurant-prepared pizza, for example, contributed 2% and 14%, respectively, of sodium from separate sources, and 24% and 65%, respectively, of sodium from pizza. On the contrary, for soups and breads/rolls, like categories that were not highlighted, sodium contributions of store-bought forms were higher (*P* < 0.05) and those of restaurant-prepared forms were lower (*P* < 0.05) than the average. For example, store-bought and restaurant-prepared soups contributed 6% and 2%, respectively, of sodium from separate sources and 78% and 14%, respectively, of sodium from soups.

**TABLE 1:**
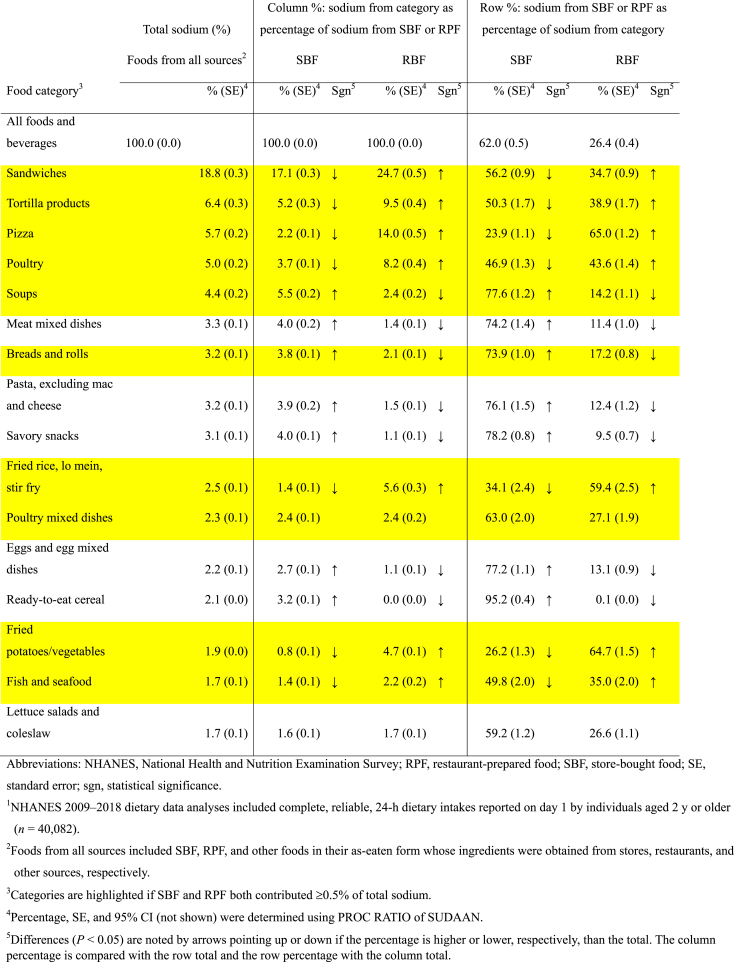
Percentages of sodium contributed by top-ranked categories of foods from all sources, SBF, and RPF in their as-eaten form: NHANES 2009–2018^1^.

**FIGURE 2:**
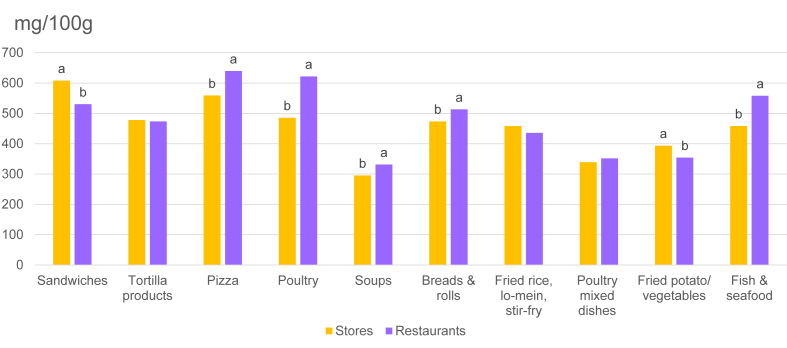
Sodium content (mg/100 g) of foods from stores and restaurants in selected categories of foods in their as-eaten form: NHANES 2009–2018 (*n* = 40,082). Differences (*P* < 0.05) are noted by a and b. Categories were selected if foods from stores and foods from restaurants both contributed ≥0.5% of total sodium.

Compared with the sodium content of store-bought forms, the sodium content of restaurant-prepared pizza, poultry, soups, fish/seafood, and breads/rolls was higher (*P* < 0.05), and the sodium content of restaurant-prepared sandwiches and fried potatoes/vegetables was lower (*P* < 0.05) (Figure 2). Data in Supplemental Table 4 demonstrate that the product of the sodium content (milligrams per 100 g) and food intake (100 grams per day) estimates equals mean sodium intake (milligrams per day).

The magnitude of the sodium contributions of subgroups shows that relatively high sodium content values have a greater influence on determining the category’s sodium content when foods are more widely consumed (Table 2). For example, lunchmeat sandwiches contain more sodium than burgers and contributed the highest percentage of intake among store-bought sandwiches, while burgers were the highest intake contributors among restaurant-prepared sandwiches. Therefore, the store-bought sandwich category composite contains more sodium than the restaurant-prepared sandwich category composite.

**TABLE 2:** The sodium content ratio, food intake percentage, and consumption-weighted sodium contribution of SBF and RPF in selected categories and subgroups of foods in their as-eaten form: NHANES 2009–2018^1^.

Food category and subgroups^2^	Sodium content^3^ (mg/100 g)			Ratio of subgroup Intake to category intake^3^ (%)			Consumption-weighted sodium contribution^4^ (mg/d)	
	SBF	RPF		SBF	RPF		SBF	RPF
Category 1: sandwiches	608	530	↓	100.0	100.0		608	530
								
Lunchmeat sandwiches	733	524	↓	29.8	17.2	↓	218	90
Cured-meat sandwiches	876	665	↓	6.6	7.3		57	49
Beef/pork sandwiches	490	434	↓	5.0	7.2	↑	24	31
Chicken/turkey sandwiches	433	537	↑	6.1	13.7	↑	26	74
Fish sandwiches	442	431		4.0	3.2		18	14
Burger sandwiches	475	488		11.8	34.8	↑	56	170
Hotdog/sausage sandwiches	739	663	↓	10.4	2.1	↓	77	14
Breakfast sandwiches	551	637	↑	12.9	12.9		71	82
Vegetarian sandwiches	442	424		13.5	1.4	↓	60	6
								
Category 2: tortilla products	478	473		100.0	100.0		478	473
								
Taco/tostada salad	334	317		3.9	6.0		13	19
Tacos/burritos	487	500		58.1	66.1	↑	283	330
Other tortilla products	478	443		38.0	27.9	↓	182	124
								
Category 3: pizza	559	640	↑	100.0	100.0		559	640
								
Pizza with meat	573	652	↑	72.3	75.6		414	493
Pizza without meat	506	601	↑	20.6	24.2		104	146
Pizza rolls	663	630	↓	4.9	0.1	↓	33	0
Lunchable flatbread pizza	353	463	↑	2.2	0.1	↓	8	1
								
Category 4: poultry	485	622	↑	100.0	100.0		485	622
								
Chicken/turkey patties/nuggets	633	678	↑	10.2	26.9	↑	65	183
Breaded/fried chicken, not reformed	514	659	↑	34.4	51.9	↑	177	342
Chicken/turkey, not breaded/fried	440	458		55.4	21.2	↓	244	97
								
Category 5: soups	295	331	↑	100.0	100.0		295	331
								
Broth and stock	248	362	↑	0.7	0.4		2	1
Shelf-stable soup	295	312		30.1	37.5		89	117
Home-recipe soup	273	308		12.2	10.4		33	32
Soup, form not specific	302	349	↑	54.9	51.8		166	181
Instant/dry mix soup	256	-		2.1	0.0	↓	5	-
								
Category 6: breads and rolls	473	513	↑	100.0	100.0		473	513
								
Bread, white/wheat	491	518	↑	57.2	28.9	↓	281	150
Rolls, white/wheat	492	503		7.3	14.3	↑	36	72
Rye bread/rolls	592	573		1.4	1.7		8	10
Flatbread	374	456	↑	7.6	9.2		28	42
Garlic/cheese bread/rolls/sticks	577	597		6.5	25.5	↑	38	152
Bagels	422	424		15.0	18.3		63	77
English muffins	375	361		4.6	1.0	↓	17	4
Fried dough	375	538		0.4	1.1		1	6
								
Category 7: Fried rice, lo mein, stir fry	458	436		100.0	100.0		458	436
								
Meat/poultry mixed with soy sauce	422	436		39.5	46.9		167	204
Fish/shellfish mixed with soy sauce	435	448		3.7	4.4		16	20
Rice/noodles mixed with soy sauce	439	427		41.4	45.3		182	193
Meatless mixed dish with soy sauce	604	530		15.4	3.5	↓	93	18
								
Category 8: poultry mixed dishes	339	352		100.0	100.0		339	352
								
Mixed fried poultry dishes	415	429		5.4	5.0		22	22
Mixed poultry, not breaded/fried	339	331		51.7	31.9	↓	175	105
Chicken/turkey pot pies, frozen meals	342	399	↑	18.9	3.2	↓	65	13
Chicken/turkey salad	319	354		24.0	60.0	↑	77	212
								
Category 9: fried potatoes/vegetables	393	354	↓	100.0	100.0		393	354
								
Fried vegetables, e.g., onion rings	304	476	↑	3.7	4.1		11	20
French fries, white/sweet, without topping	376	312	↓	30.1	71.6	↑	113	224
French fries, white/sweet, with topping	543	522		2.7	4.1		14	22
Home fries	331	349		36.2	5.4	↓	120	19
Hash browns	489	455		20.0	12.2	↓	98	56
Tater tots	499	575	↑	7.4	2.5	↓	37	14
								
Category 10: fish and seafood	458	557	↑	100.0	100.0		458	557
								
Fish, not breaded/fried	423	409		52.4	30.6	↓	222	125
Breaded/fried fish	416	425		26.1	28.8		109	122
Shellfish, not breaded/fried	652	643		12.8	21.0		83	135
Breaded/fried shellfish	590	921	↑	3.0	18.6	↑	18	172
Raw fish/seafood	81	187		0.2	0.7		0	1
Canned fish/seafood	331	—		4.7	0.0	↓	15	—

Abbreviations: NHANES, National Health and Nutrition Examination Survey; RPF, restaurant-prepared foods; SBF, store-bought foods; SE, standard error.

1 NHANES 2009–2018 dietary data analyses included complete, reliable, 24-h dietary intakes reported on day 1 by individuals aged 2 y or older (*n* = 40,082). SBF and RPF are defined as foods in their as-eaten form whose ingredients were obtained from stores and restaurants, respectively.

2 Categories are selected if SBF and RPF both contributed ≥0.5% of total sodium.

3 The sodium content ratio, percentage, SE (not shown), and 95% CI (not shown) were determined using PROC RATIO of SUDAAN. Differences (*P* < 0.05) are noted by arrows pointing up or down if the RPF estimate is higher or lower, respectively, than the SBF estimate.

4 Consumption-weighted sodium contributions of subgroups represent the sodium intake (mg/d) contributed by proportions of a 100-g portion of a food category composite. The summed total of subgroup contributions represents the sodium intake (mg/d) contributed by the 100-g portion.

The mean sodium intake contributions and reductions of sodium intake from subgroups of foods were ranked to help set priorities for sodium-reduction efforts (Table 3). Color-coded tiers include groups of the highest (red), second highest (orange), third highest (yellow), and lowest (white) ranked sodium intake contributions and ranked sodium intake reductions, and no rank was shown if the estimates were minimal.

**TABLE 3:**
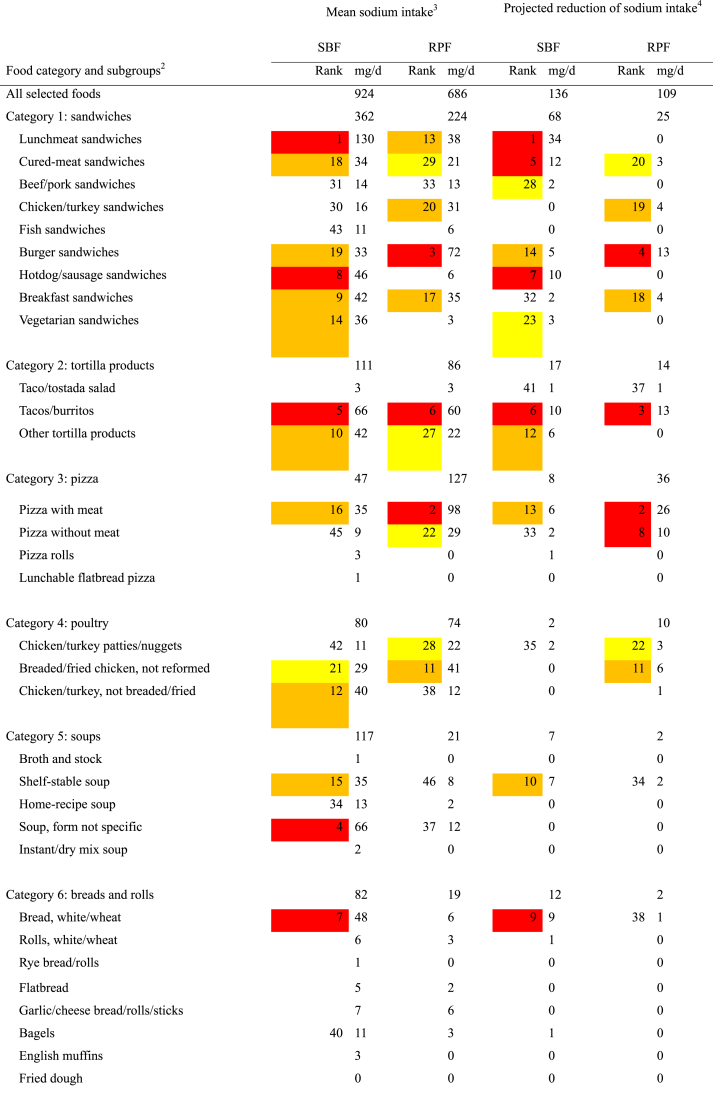

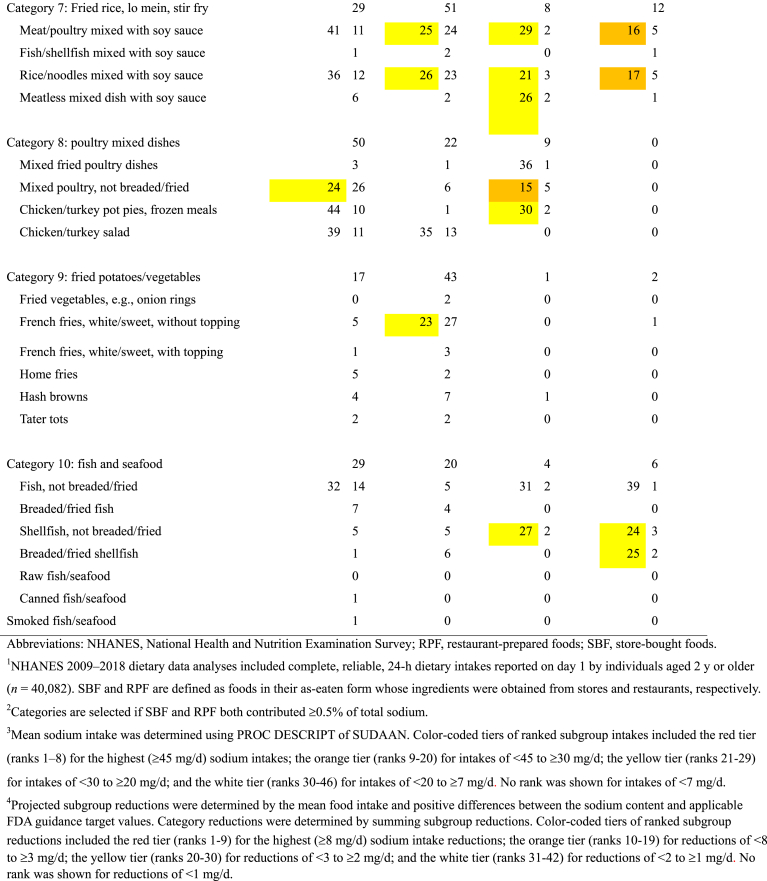
Mean sodium intake and projected sodium intake reductions that would be achieved by meeting FDA targets for SBF and RPF in selected categories and subgroups of foods in their as-eaten form: NHANES 2009–2018^1^.

In general, meeting FDA targets for the largest sodium contributors was found to have the greatest projected impact on sodium intake reduction. For example, the highest (red) tiers included both contributions and projected reductions of sodium intake from the following subgroups of foods: store-bought lunchmeat sandwiches, restaurant-prepared pizza with meat, restaurant-prepared burger sandwiches, store-bought and restaurant-prepared tacos/burritos, store-bought hotdog/sausage sandwiches, and store-bought white/wheat bread. The summed total of ranked (highest to lowest) sodium intake reductions achieved by meeting FDA targets for subgroups in each of the 4 tiers would be 138, 54, 27, and 17 mg/d, respectively, and the total sodium intake reduction achieved by meeting FDA targets for all subgroups would be 245 mg/d or 15% of the sodium intake contributed by the 10 selected categories of store-bought and restaurant-prepared foods.

## Discussion

This study showed sodium content reductions targeting top sodium contributors would most effectively reduce sodium intake from United States diets. In this study, categories included both combined and single-code foods; however, in most previous research, components of food combinations had been disaggregated and classified into separate food categories [6,7,21].

Although food grouping changes had some effect, differences in the ranked food category contributions of sodium between 2007–2008 [6] and 2013–2014 [7] were likely due to changes in food coding procedures involving the development of single food codes for some sandwiches and burritos (Table 4) [6,7,21,22]. In earlier WWEIA cycles, sandwich codes were mostly for restaurant-prepared sandwiches, and sandwich codes added later were mostly for home-prepared sandwiches [16]. Inconsistent disaggregation could potentially bias comparisons limited to store-bought and restaurant-prepared foods reported using single codes. In this study, none of the combination foods were disaggregated, and food classification was consistent over time.

**TABLE 4:** Top-ranked food category contributors of sodium found by 5 NHANES studies^1^.

Food category^2^	2007–2008^3^	2013–2014^4^^,^^5^	2015–2016^5^, ^6^, ^7^	2017–2018^5^, ^7^, ^8^	2009–2018^9^
	(*n* = 7227)	(*n* = 8067)	(*n* = 7976)	(*n* = 7081)	(*n* = 40,082)
Sandwiches	6	3			1
Deli meat sandwiches			1		
Burgers			9	10	
Pizza	3	2	2	1	3
Burritos and tacos		6	3	5	2
Soups	5	5	4	4	5
Savory snacks and crackers	10	7	5	6	9
Poultry, excluding nuggets and tenders	4	8	6	7	4
Pasta, excluding macaroni and cheese	8	12	7	9	8
Breads and rolls	1	1		2	7
Cold cuts and cured meats	2	4		3	
Cheese	7	9		8	
Eggs and omelets		10	10		12
Meat mixed dishes	9	11		11	6
Bacon, frankfurters, sausages		13		13	
Vegetables, excluding white potatoes			8	14	
Fried rice, lo mein, stir fry mixtures					10
Poultry mixed dishes					11
Cookies, brownies, cakes, pies				12	
Ready-to-eat cereal					13

Abbreviations: NHANES, National Health and Nutrition Examination Survey; WWEIA, What We Eat in America.

1 NHANES dietary data analyses included complete, reliable, 24-h dietary intakes reported on day 1 by individuals aged 2 y or older.

2 In the 2007–2008, 2013–2014, and 2017–2018 studies, if 15 types of food combinations were represented using multiple food codes, their components had been classified into separate WWEIA food categories. In the 2015–2016 study, disaggregated components of sandwiches were grouped together and reclassified to 1 of the 7 sandwich categories, while components of other food combinations remained disaggregated. In this study, disaggregated components of all 15 types of combinations were grouped together and reclassified to a WWEIA food category that represents the combined food. Supplemental Table 1 summarizes the correspondence to WWEIA food categories of 104 food categories used in the 2007–2008 study and this study. Some of the 104 food categories were grouped together or split apart to form 109 categories used in the 2013–2014 study, and 87 categories used in the 2015–2016 and 2017–2018 studies.

3 CDC, 2012 [6]

4 Quader et al., 2017 [7]

5 Burritos and tacos, nachos, and other Mexican mixed dishes were split into 3 categories. Savory snacks and savory crackers were grouped together. Chicken (whole pieces), turkey, duck, other poultry, and chicken patties, nuggets, and tenders were split into 2 categories. Bacon, frankfurters, and sausages were grouped together. Fried rice, lo/chow mein, stir fry, and soy-based sauce mixtures were split into 2 categories.

6 Woodruff et al., 2020 [22], included individuals aged 1 y or older.

7 Sandwiches were split into 7 categories. Red and orange vegetables, dark green vegetables, lettuce and lettuce salads, starchy vegetables (excluding white potatoes), and other vegetables were grouped together. Cookies, brownies, cakes, and pies were grouped together.

8 Ahmed et al., 2023 [21].

9 This study.

In this study and in a study by Sebastian et al. [23], sandwiches included both combined and single-code sandwiches; and in both studies, their sodium contribution was 19%. In contrast, sodium contributed by single-code sandwiches alone was 4%–6% in the 2007–2008 and 2013–2014 NHANES studies [6,7] (Table 4). Top-ranked sandwich components found in these studies [6,7] were not found to be top sodium contributors when combined and single-code sandwiches were grouped together in the NHANES 2015–2016 study by Woodruff et al. [22]. Categories used by both Woodruff et al. [22] and Ahmed et al. [21] included sandwiches split into 7 categories. When combined sandwiches were included in sandwich categories, lunchmeat sandwiches and burgers were the first and ninth highest sodium contributors, respectively [22]; however, only burgers contributed ≥2% of dietary sodium when combined sandwiches were not included [21].

This study confirmed previous findings that restaurant-prepared foods contributed ∼25% of total sodium [6,7,24]. Previous research showed restaurant food consumption was associated with intakes of sandwiches, pizza, Mexican mixed dishes, Asian mixed dishes, poultry, and fried potatoes [25], and sodium contributions from restaurant-prepared forms of these foods were higher than the average. However, comparisons to store-bought forms showed the sodium content of restaurant-prepared sandwiches and fried potatoes was lower. Although restaurant-prepared soups and breads contained more sodium, their intakes and sodium contributions were lower.

The FDA had determined sodium content as a ratio of sales-weighted sodium contributions and total sales [5]. Similarly, in this study, sodium content was determined from consumption-weighted sodium contributions to show that FDA targets would be most effectively achieved by reducing sodium from widely consumed foods. This demonstration showed sales-weighted targets could be achieved not only by food reformulation; they could also be achieved by decreasing supply and demand for high-sodium foods and/or by promoting demand for lower-sodium foods.

Although sodium content comparisons of store-bought with restaurant-prepared foods were limited to 10 selected categories, comparisons with FDA targets could be extended to other categories including packaged foods that were more frequently obtained from stores than restaurants. This approach could also be extended to a more granular level to identify smaller groups of the targeted foods that contribute the most sodium. Analyses of sodium contributed by different types of luncheon-meat, chicken, or burger sandwiches could show, for example, that sodium reductions would be most effectively achieved by replacing ham with sliced turkey, replacing fried fillets with grilled chicken, or eliminating bacon from cheeseburgers.

In summary, reductions of sodium in widely consumed foods, such as luncheon-meat sandwiches and restaurant-prepared pizza, were found to have the greatest impact on reducing sodium intake by the United States population. These findings could be used by restauranteurs, food manufacturers, policy makers and regulators, and clinical practitioners to inform sodium-reduction efforts. Guidance could suggest that restauranteurs, food manufacturers, and consumers could substitute or eliminate components of foods, such as meat toppings or sauces, which contribute the most sodium; and the demand for lower-sodium options, such as grilled chicken sandwiches, could be increased through marketing promotions and consumer education. The identification of top sodium contributors could help set priorities to further reduce sodium intake, reduce the prevalence of hypertension, and improve public health.

## Author contributions

The authors’ responsibilities were as follows – DRK: designed the research and conducted the research by analyzing the data and performing statistical analyses; DRK, PMG: wrote the article; DRK: had primary responsibility for final content; and both authors: read and approved the final manuscript.

## Conflicts of interest

DRK reports that financial support and article publishing charges were provided by Institute for the Advancement of Food and Nutrition Sciences. The other author reports no conflicts of interest.

## Funding

This work was supported by the Institute for the Advancement of Food and Nutrition Sciences (IAFNS) Sodium in Food & Health Implications Committee. IAFNS is a nonprofit science organization that pools funding from industry and advances science through the in-kind and financial contributions from private and public sector members. IAFNS had no role in the design, analysis, interpretation, or presentation of the data and results.

## Data availability

The data sets analyzed in this study are available online in the Centers for Disease Control and Prevention repository at: http://www.cdc.gov/nchs/nhanes/. Upon request, authors are willing to help others replicate or conduct similar analyses.
