# Supplementary material for: Sodium Content and Sodium Intake Contributions of Store-Bought and Restaurant-Prepared Foods in Their As-Eaten Form: National Health and Nutrition Examination Survey, 2009–2018
Source: Curr Dev Nutr. 2024 Sep 2;8(10):104455. doi: 10.1016/j.cdnut.2024.104455 (PMC11480230; doi:10.1016/j.cdnut.2024.104455)
Supplement: multimedia component 1 [file mmc1.docx]

Sodium Content and Sodium Intake Contributions of Store-Bought and Restaurant-Prepared Foods in Their As-eaten Form: NHANES, 2009-2018

Debra R. Keast, PhD

SUPPLEMENTAL MATERIALS

SUPPLEMENTAL METHODS

Statistical analysis

The sodium content (mg/100g) ratio as well as mean sodium intake (mg/d), mean food intake (g/d), and population proportions (%), were determined for selected categories and subgroups of the selected categories of foods obtained from stores and restaurants. Sample-weighted data were analyzed using SUDAAN Release 11.0.3 (RTI International, 2018) to adjust the standard errors of the mean and ratio estimates for the sample design. PROC DESCRIPT of SUDAAN was used to determine mean sodium intake (mg/d), mean food intake (g/d), and standard errors of the mean estimates. PROC RATIO of SUDAAN was used to determine population proportions (% of total sodium), the sodium content (mg/100g) ratio, and standard errors of the ratio estimates. Confidence intervals were determined from the standard errors of the mean and ratio estimates to test the statistical significance of differences.

Subgroup contributions

Like mean sodium intake (mg/d), the consumption-weighted subgroup contribution (mg/d) is equivalent to the product of the sodium content (mg/100g) and mean food intake estimates that had been determined using SUDAAN. In this case, however, food intakes contributed by subgroups were expressed as percentages of the category intake. Because a subgroup’s sodium content (mg/100g) ratio represents the sodium intake (mg/d) contributed by a 100g portion of a food subgroup composite, with subgroup intakes expressed as proportions of the category intake, the consumption-weighted sodium contribution of the subgroup then represents the sodium intake (mg/d) contributed by a proportion of a 100g portion of the food category composite. The summed total of the subgroup contributions then represents the sodium intake (mg/d) contributed by the 100g portion.

USDA performs similar calculations using the FNDDS to determine the nutrient content of food composites. For example, if consumption of a sandwich with no further specification of the sandwich type was reported in the dietary interview, the sodium content, i.e., sodium intake from each 100-gram portion of the “sandwich, not further specified” composite. could be determined by summing the consumption-weighted sodium contributions of each sandwich type. A subgroup’s proportion of the summed consumption-weighted contributions is equivalent to the percentage of sodium intake (mg/d) from the category that was contributed by sodium intake (mg/d) from the subgroup.

Subgroup correspondence to FDA guidance categories

As shown in Supplemental Table 2, subgroups of the selected categories were mapped to the FDA guidance categories to identify the appropriate sodium content targets that apply to the foods in each subgroup. The sodium content of foods from separate sources was determined because FDA included separate target values for packaged foods and restaurant foods.

Separate estimates were also determined if separate target values applied to different groups of foods that had been classified to one food category subgroup. For example, in the burger sandwich subgroup, separate estimates were determined for beef burgers without cheese, beef burgers with cheese, turkey/chicken burgers, vegetarian burgers without cheese, and vegetarian burgers with cheese because separate targeted foods included hamburgers without cheese, hamburgers with cheese, poultry/fish-based sandwiches, vegetarian sandwiches without cheese, and vegetarian sandwiches with cheese, respectively.

Because many foods targeted by FDA were prepared foods, such as sandwiches or meats served with sauces, the sodium content of foods in their as-eaten form was determined. However, packaged foods, such as breads/rolls and packaged French fries, hash browns and tater tots, were separated from additions to apply the FDA guidance to packaged foods without additions.

Projected sodium intake reductions

Sample-weighted mean food intake (g/d) and sodium content (mg/100g) estimates generated by SUDAAN analyses were used to calculate projected sodium intake reductions that would be achieved by meeting each subgroup’s sodium content target. Projected sodium intake reductions were determined by the product of mean food intake and positive differences between the sodium content and applicable target values. If the sodium content ratio was less than the target and the difference was negative, the projected reduction was set to zero. Category reductions were determined by summing the subgroup reductions.
